# New Records and New Species of Dacnusini (Hymenoptera: Braconidae, Alysiinae) Based on Morphological and Molecular Evidence

**DOI:** 10.3390/insects15110835

**Published:** 2024-10-24

**Authors:** Jiachen Zhu, Cornelis van Achterberg, Xuexin Chen, Pu Tang

**Affiliations:** 1 Zhejiang Key Laboratory of Biology and Ecological Regulation of Crop Pathogens and Insects, Zhejiang University, Hangzhou 310058, China; zjchen828@163.com (J.Z.); xxchen@zju.edu.cn (X.C.); 2 Institute of Insect Sciences, College of Agriculture and Biotechnology, Zhejiang University, Hangzhou 310058, China; kees@vanachterberg.org; 3 Ministry of Agriculture Key Laboratory of Molecular Biology of Crop Pathogens and Insects, Zhejiang University, Hangzhou 310058, China

**Keywords:** Dacnusini, *Laotris*, *Victorovita*, *Coloneura*, new record, DNA barcode, taxonomy, keys

## Abstract

Dacnusini is a species-rich tribe in the subfamily Alysiinae, with most species exclusively serving as parasitoids of leaf-mining Diptera (Agromyzidae). The number of genera discovered in China remains limited, which is apparently insufficient considering the global diversity of species and genera within this tribe, particularly given the vast and ecologically diverse landscapes of China. In the present study, three new record genera, *Victorovita* Tobias, *Coloneura* Foerster, and *Laotris* Nixon, were documented for the first time in China. In addition, the species delimitation approach and haplotype network analyses based on the COI sequences, combined with morphological evidence, were employed to delimit species. The findings indicated three new species: *Laotris glabella* sp. nov., *Laotris aethidentata* sp. nov., and *Victorovita aequalis* sp. nov. Additionally, K2P divergences showed no overlap between intra- and interspecific genetic distances in the *Laotris* and *Victorovita* species. Detailed descriptions for new species and keys to the species of *Laotris* and *Victorovita* are provided in this paper, along with the documentation of two new species records for China: *Victorovita caudata* (Szépligeti, 1901) and *Coloneura stylata* Foerster, 1863.

## Simple Summary

The tribe Dacnusini is a small braconid parasitoid wasp within the subfamily Alysiinae (Hymenoptera: Braconidae), consisting of 31 valid genera and more than 877 species worldwide. Most members are exclusively endoparasitoids of leaf-mining Diptera (Agromyzidae), and several species have been utilized in commercial biological control programs, underscoring their essential role in natural pest management. However, due to low interspecific variability and significant morphological convergence, distinguishing between genera and species within the genera in Dacnusini presents a challenge. In this study, we report three genera, *Victorovita* Tobias, *Coloneura* Foerster, and *Laotris* Nixon, that were discovered in China for the first time. By integrating DNA barcoding and morphological evidence, three new species were identified through multiple species delimitation methods. Additionally, two new species records from China are reported, and identification keys for species of *Laotris* Nixon, 1943 are provided.

## 1. Introduction

The subfamily Alysiinae (Hymenoptera: Braconidae) is a highly diverse group, comprising over 2450 described species worldwide [1]. It is distinguished by outwardly directed exodont mandibles that do not meet when closed, featuring three to five teeth [2,3]. Alysiinae includes two tribes: Alysiini and Dacnusini. A key difference between them is the absence of vein r-m in Dacnusini. Despite having nearly the same number of species, Dacnusini includes fewer genera (31 compared to 76 in Alysiini) [3,4]. Griffiths [5,6,7,8] revised the generic classification of Dacnusini and implemented phylogenetic methods, suggesting that Dacnusini is monophyletic, which recent molecular research confirmed, while Alysiini is paraphyletic [9]. Most Dacnusini species are exclusively parasitoids of leaf-mining Diptera (Agromyzidae), and several species are commercially employed in biological control programs.

The genus *Victorovita* Tobias, 1985, comprises only three known species and is distributed in East Palaearctic and Europe [4]. It can be recognized by two distinctly developed teeth, a narrow ventral lamella and a flat clypeus. Tobias [10] established the genus and designated *V. genalis* Tobias, 1985 as the type-species. *V. caudata* was originally described by Szépligeti [11] but was synonymized with *Amyras clandestina* (Haliday) by Shenefelt [12]. Papp [13] revalidated *V. caudata* Szépligeti as a valid species and synonymized *V. genalis* Tobias as a junior synonym. Perepechayenko [14] disagreed with this synonymy (without giving arguments) and described a new species: *V. minuta*. Little is known about the biology of *Victorovita*.

*Coloneura* Foerster, 1863 is a small genus primarily found in the Palaearctic region, with fifteen known species [4]. It is distinguished by the open apico-posterior first subdiscal cell of the fore wing and the absence of vein CU1b. This genus was proposed by Foerster [15] based on *Coloneura stylata* Foerster. New species were later described by Nixon [16,17], Griffiths [6,7], Tobias and Jakimavicius [18], and Tobias [19]. Griffiths [5,7] synonymized several genera under *Coloneura*, while van Achterberg [20] revised the genus and described a new subgenus, *Coloneurella*, which was later elevated to genus rank. The biology of *Coloneura* is reported in a few records, mainly as parasitoids of *Phytomyza*, *Liriomyza*, and *Phytoliriomyza* (Agromyzidae) [7].

*Laotris* Nixon, 1943 is another small Dacnusini genus with only four described species, primarily distributed in the Palaearctic region [4,21]. Nixon [16] established this genus for *L. striatula* (Haliday), originally described in *Alysia* Latreille by Haliday [22], later moved to *Dacnusa* by Marshall [23]. Griffiths [24] described *L. rupestris*, distinguished by its smooth metapleuron. Tobias [19] discovered *L. minuscularia* in Russia. Godfray [21] described *L. luzulae*, the fourth species, based on morphology and molecular evidence. Griffiths [5] suggested that *Laotris* might belong to the *Coelinius* group by sharing the derived characters of the striate second metasomal tergite and a fourth small tooth on the mandible. van Achterberg [25] and Zhang et al. [26] agreed with this classification. The biology of *Laotris* is clear, and *L. striatula* is a parasitoid of *Cerodontha luctuosa* mining *Juncus effusus* (Juncaceae), while *L. rupestris* is parasitoid of *Cerodontha* sp. mining *Carex sempervirens* (Cyperaceae) [21,24,27]. *L. luzulae* was reared from *Cerodontha silvatica* mining *Luzula sylvatica* (Juncaceae) [21].

For this paper, we thoroughly examined thousands of Dacnusini specimens collected from various provinces in China. Molecular phylogenies based on COI sequence region were reconstructed using the maximum likelihood method. Two species delimitation approaches, along with haplotype network analyses, were applied to investigate the intra- and interspecific variation.

## 2. Materials and Methods

### 2.1. Taxon Sampling

The examined specimens were collected by sweeping net and Malaise traps and stored in 99% alcohol. Later, they were glued onto card points for better identification. All the specimens were deposited in the Parasitic Hymenoptera Collection, Institute of Insect Sciences of the Zhejiang University (ZJUH).

### 2.2. Specimen Examination

For the recognition of the subfamily Alysiinae, see the studies by van Achterberg [2,28] and van Achterberg [29]. For additional references, see Yu, van Achterberg, and Horstmann [4]. The terminology and measurements used follow the work by van Achterberg [2,30]. The following abbreviations are used: POL—postocellar line; OOL—ocular-ocellar line, measured from ocellus directly to eye; OD—maximum diameter of lateral ocellus. The medial length of the first tergite is measured from the apex of the adductor to the apex of the tergite. Descriptions and measurements were made using a Leica M125 stereomicroscope. Photographs were made using a Keyence VHX-7000 digital microscope (Keyence Corporation, Osaka, Japan), and the photos were slightly processed (mainly cropped and with a modification made to the background) in Photoshop CC.

### 2.3. Molecular Analysis

DNA was extracted using the QIAamp DNA Mini Kit (Qiagen, Hilden, Germany) and followed the non-destructive methods [31]. Amplification of 658 bp fragment of COI barcode region [32] was carried out using the primers LCO-1490 [33] and HCO-700ME [34]. PCR amplifications were performed using 2× Phanta Max Master Mix (Dye Plus) (Vazyme Biotech Co., Nanjing, China) DNA polymerase in a 20 μL mix containing 1 μM of each primer, 10 units of 2× Phanta Max Master Mix (Dye Plus), and 3 μL of cDNA. The PCR cycling protocols were performed as follows: 94 °C for 1 min and a five-cycle preamplification (30 s for 94 °C, 40 s for 45 °C, and 1 min for 72 °C), followed by 35 cycles of 30 s for 98 °C, 40 s for 55 °C, and 1 min for 72 °C, and a final extension of 2 min for 72 °C.

We additionally downloaded Barcode Index Number (BIN) data (ACD0274) of *Dapsilarthra sylvia* as an outgroup and all BINs of *Laotris* Nixon for analysis. The BIN system was established on the Barcode of Life Data (BOLD, https://boldsystems.org/ (accessed on 21 October 2024) platform to register OTUs delineated by the RESL algorithm [35]. The BOLD system assigns a BIN to all barcode records. However, it is important to note that BIN codes can change over time as more specimens are added or as species are reassigned. Tracking these changes is time-consuming, and there is currently no formal method for tracing specimens through changing BINs [36]. All sequences from this study have been deposited in both the GenBank and BOLD databases (Table S1). Geneious Prime 2023.2.1 was used for sequence editing. Muscle v5.1 was used for alignment using default settings [37]. Sequence divergences for intraspecific and interspecific pairwise genetic distances were computed based on the Kimura-2 parameter (K2P) model [38] in MEGA 11 (Table S2). Maximum-likelihood (ML) analyses were performed using IQ-TREE v2.0 [39] and the best-fitting substitution model was identified using ModelFinder implemented in IQ-TREE (MFP). FigTree v1.4.3 was utilized to visualize and illustrate the inferred phylogenetic trees.

The Automatic Barcode Gap Discovery (ABGD) analysis was employed for species delimitation. ABGD is a method used to delimit species based on barcode gap, which is the difference between intra-specific and inter-specific variation, and automatically partitions the sequences into candidate species without requiring an a priori threshold. The ABGD analysis was conducted via a web interface (https://bioinfo.mnhn.fr/abi/public/abgd/abgdweb.html, accessed on 28 September 2024), using the K2P model to classify species based on genetic distances. The relative gap width (X) was set to 1.0, and the remaining parameters were set to default [38]. A haplotype network was constructed using the TCS method implemented in the software PopART v.1.7 [40].

## 3. Results

### 3.1. Genetic Distances and Species Delimitation

Species of *Laotris* come from different regions. *Laotris* (ADV6100) specimens were all collected from Canada, whereas *Laotris luzulae* (AEO8807) and *Laotris striatula* (AEO8806) were from England. Additionally, one specimen of *Laotris* (ACY7573) was sampled from England, while another came from Colorado, USA. The Kimura-2-parameter (K2P) genetic distances of interspecies and intraspecies were summarized in Table 1. *Laotris glabella* sp. nov. exhibited the smallest average interspecific distance (0.045) with *Laotris luzulae* (AEO8807), which is notably greater than the maximum intraspecific distance observed within the *Laotris* genus (0.02). Similarly, the interspecific genetic divergence between *Victorovita aequalis* sp. nov. and *Victorovita caudata* (0.044) exceeds their intraspecific divergence (0–0.013).

**Figure 1:**
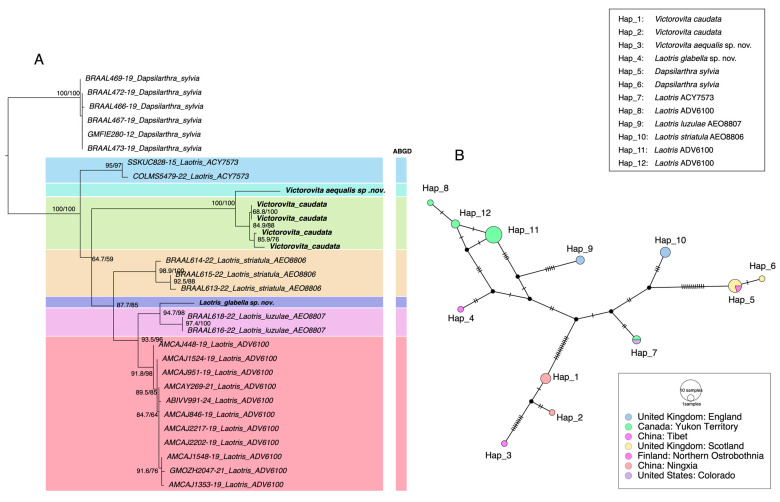
Comparison of species delimitation results using different methods. (**A**) Maximum likelihood (ML) phylogenetic tree inferred from the COI barcode region, highlighting the results from ABGD delimitation analyses. The number on the left of each node indicates SH-aLRT support values, while the number on the right represents ultrafast bootstrap support (BS); only values above 50 were shown. (**B**) TCS haplotype networks for the various species, where each hatch mark represents a mutational step between adjacent alleles. The color of each circle corresponds to the geographic origin of the sequence (refer to the inset figure legend), and the size of each circle is proportional to the haplotype frequency.

Species delimitation results from the two approaches are summarized in Figure 1. The ABGD analysis divided COI sequences of the Dacnusini group into seven molecular operational taxonomic units (MOTUs), consistent with both morphology and phylogenetic analysis (Dataset S1). The initial partition and recursive partition under the K2P model are shown in Figure S1. Additionally, a haplotype network was constructed for further analysis. The TCS haplotype network revealed 12 distinct haplotypes (Hap_1–Hap_12) distributed across different geographic regions and divided 7 Dacnusini morphological species into corresponding genetic groups (Figure 1B). *Laotris glabella* sp. nov. and *Victorovita aequalis* sp. nov. displayed far mutation steps from related species, which aligns with the findings from ABGD and phylogenetic analyses. This study provided the first sequence data for the genus *Victorovita*. Using both the BOLD Identify Tool and the Neighbor-Joining (NJ) tree construction tool within the BOLD system, we assessed the genetic relationships between *Victorovita* and known species in the database. The identify tool indicated that *Victorovita* shares 92.63% sequence similarity with *Dacnusa faeroeensis*, whereas the NJ tree indicated a closer evolutionary relationship with *Synelix* species. Despite the comprehensive use of multiple methods, we could not obtain the COI sequences for *Laotris aethidentata* sp. nov. and *Coloneura stylata* Foerster.

In summary, three genera, *Victorovita* Tobias, 1985, *Coloneura* Foerster, 1863, and *Laotris* Nixon, 1943, were first documented in China. Three new species were identified: *Laotris aethidentata* sp. nov. based on morphological evidence, and two additional species, *Laotris glabella* sp. nov., and *Victorovita aequalis* sp. nov., supported by both morphological and molecular evidence. In addition, two new record species, *Victorovita caudata* (Szépligeti, 1901) and *Coloneura stylata* Foerster, 1863 were discovered and described. Keys to known species of *Laotris* Nixon and *Victorovita* Tobias are provided in this paper.

### 3.2. Taxonomy

Laotris Nixon, 1943

*Laotris* Nixon, 1943: 30; Wharton, 1994: 635; Godfray & van Achterberg, 2024: 57. Type-species: *Alysia* (*Dacnusa*) *striatula* Haliday, 1839.

**Figure 2:**
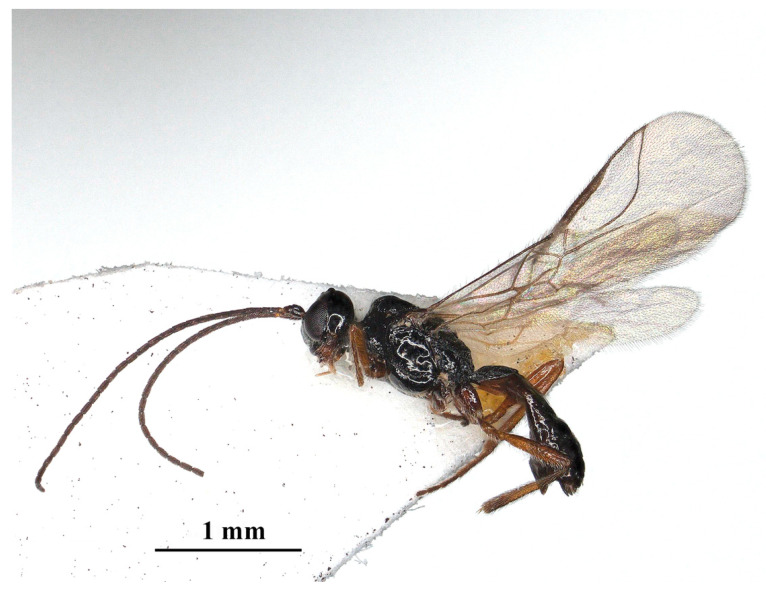
*Laotris glabella* sp. nov., ♀, holotype, habitus, lateral aspect.

**Figure 3:**
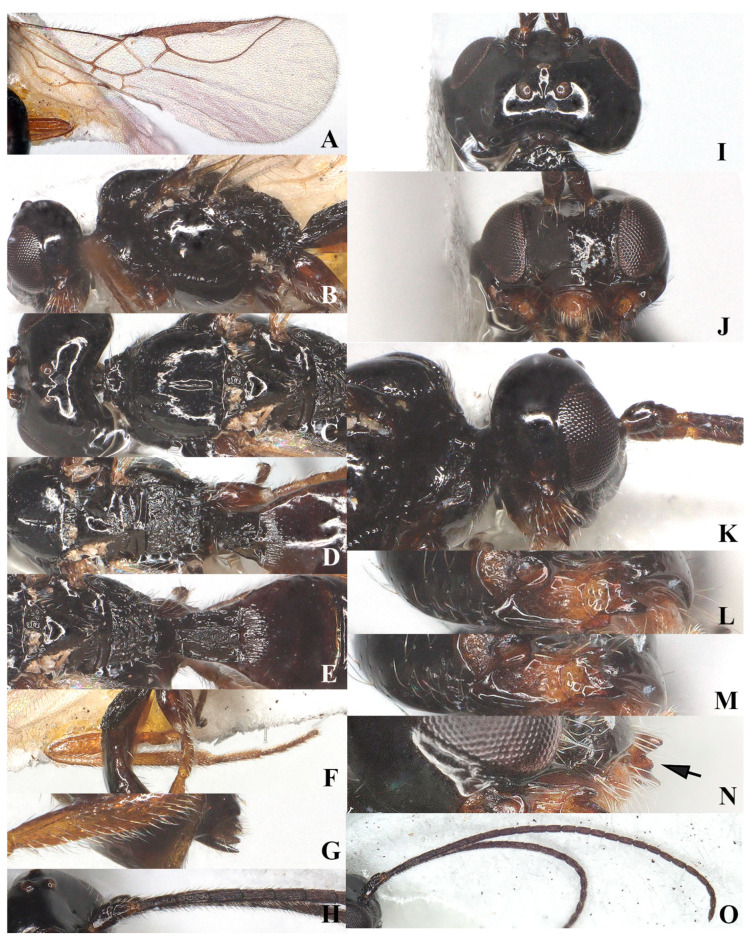
*Laotris glabella* sp. nov., ♀, holotype (**A**) wings; (**B**) mesosoma, lateral aspect; (**C**) mesosoma, dorsal aspect; (**D**) propodeum, dorsal aspect; (**E**) metasoma, dorsal aspect; (**F**) hind leg, lateral aspect; (**G**) ovipositor, lateral aspect; (**H**) basal segments of antenna, lateral aspect; (**I**) head, dorsal aspect; (**J**) head, anterior aspect; (**K**) head, lateral aspect; (**L**) mandible, full view of first and second tooth; (**M**) mandible, full view of third tooth; (**N**) mandible, full view of fourth tooth; (**O**) antenna, lateral aspect.

**Figure 4:**
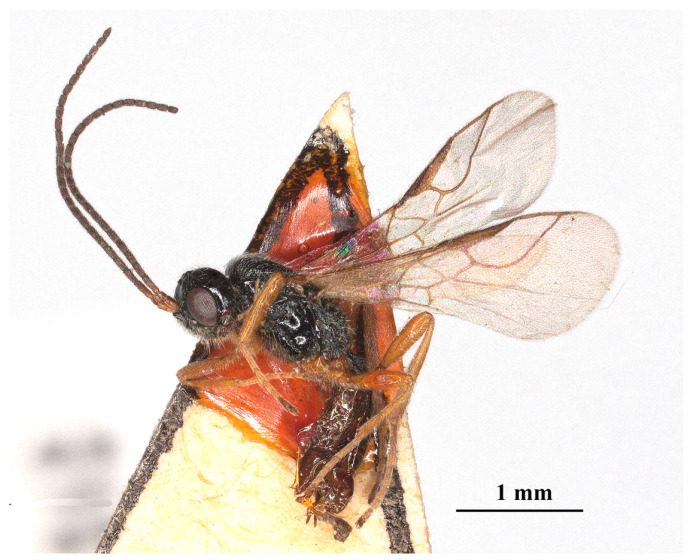
*Laotris aethidentata* sp. nov., ♀, holotype. habitus, lateral aspect.

**Figure 5:**
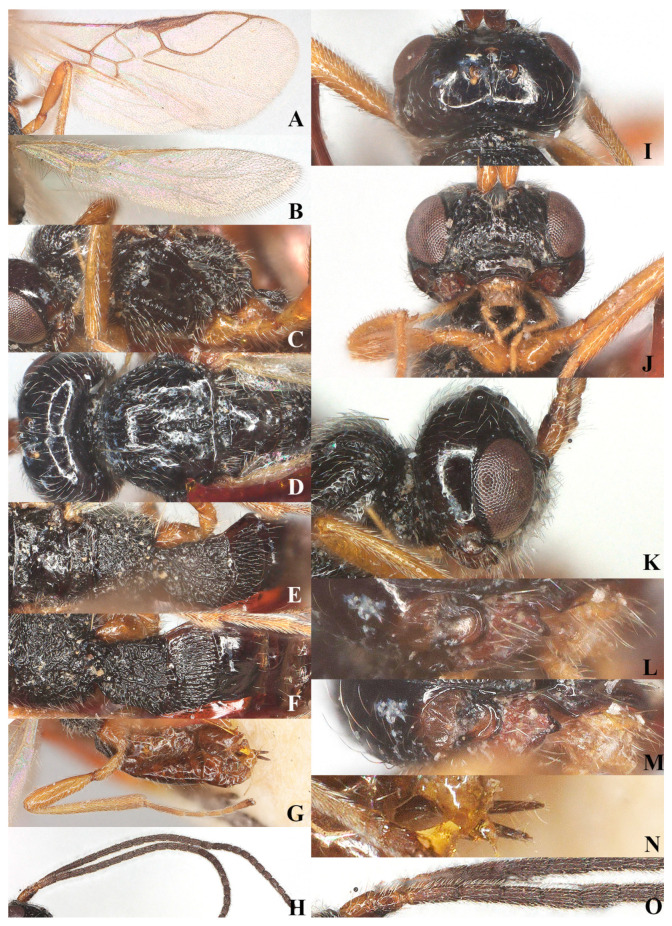
*Laotris aethidentata* sp. nov., ♀, holotype (**A**) fore wing; (**B**) hind wing; (**C**) mesosoma, lateral aspect; (**D**) mesosoma, dorsal aspect; (**E**) propodeum, dorsal aspect; (**F**) metasoma, dorsal aspect; (**G**) hind leg, lateral aspect; (**H**) antenna, lateral aspect; (**I**) head, dorsal aspect; (**J**) head, anterior aspect; (**K**) head, lateral aspect; (**L**) mandible, full view of first and second tooth; (**M**) mandible, full view of third tooth; (**N**) ovipositor, lateral aspect; (**O**) basal segments of antenna, lateral aspect.

Diagnosis: Body black (Figure 2, Figure 3, Figure 4 and Figure 5). Head transverse; eyes glabrous; clypeus smooth, distinctly protruding in lateral view, ventro-lateral corners of clypeus rather acute (Figure 3J and Figure 5J); mandible with three or four teeth (a minute fourth tooth as an outgrowth present on the ventral side of the long and acute second tooth or with an inconspicuous fourth tooth connected with a small round shaped tooth) (Figure 3L–N and Figure 5L,M); notauli distinctly crenulated, present at half of the mesoscutum; medio-posterior depression deep and elongated at half of the mesoscutum (Figure 3C and Figure 5D); metapleural pubescence virtually normally present but does not form a rosette (Figure 3B and Figure 5C); metanotum slightly protruding; first subdiscal cell distinctly closed and vein CU1b present (Figure 3A and Figure 5A); dorsope distinctly developed; second tergites of metasoma striae (Figure 3E and Figure 5F); ovipositor sheath short.

Biology: Parasitoid of Agromyzidae larvae [24].

Distribution: Eastern Palaearctic, Western Palaearctic.

Key to species of *Laotris* Nixon, 1943.

1. Antenna with only 20 segments; precoxal sulcus absent; pterostigma linear, vein r distinctly longer than width of pterostigma, vein m-cu interstitial; first tergite of metasoma brownish yellow→*L. minuscularia* Tobias, 1998.

– Antenna with 27–31 segments; precoxal sulcus present and crenulated (Figure 3B and Figure 5C); pterostigma wide and elongated, sometimes more or less parallel-sided, vein r slightly longer or shorter than width of pterostigma, vein m-cu interstitial or distinctly interstitial (Figure 3A and Figure 5A); first tergite of metasoma black (Figure 2 and Figure 4)→2.

2. Surface of second tergite entirely or at least half with longitudinal striae (Figure 5D); pterostigma robust, 4.7–5.5 times as long as its width, vein r shorter than the width of pterostigma (Figure 5A); face distinctly sculptured (Figure 5J)→3.

– Surface of second tergite only anterior 1/3–1/4 with longitudinal striae (Figure 3D,E); pterostigma slender, more or less parallel-sided, 7.0–9.3 times as long as its width, vein r slightly longer than width of pterostigma (Figure 3A); face smooth or with some rugae (Figure 3J)→5.

3. Mandible short, median length as long as its width, second tooth without small outgrowth tooth, third tooth small and rounded, connected with an inconspicuous fourth tooth, all teeth directed outward (Figure 5L,M); pterostigma wide, 4.7 times as long as its width (Figure 5A); first tergite shorter than its apical width; ovipositor projects beyond apical tergite in retracted position (Figure 5F)→*L. aethidentata* sp. nov.

– Mandible comparatively long, the median length 1.5 times its width, second tooth sharp and long, with or without a fourth small outgrowth tooth on the anterior edge, all teeth directed straightforward; pterostigma less wide, 5.5 times as long as its width; first tergite longer than its apical width; ovipositor not projecting beyond apical tergite in retracted position→4.

4. Mandible with a fourth small outgrowth tooth on anterior edge; surface of metapleuron densely sculptured; second tergite of metasoma entirely with distinct longitudinal striation→*L. striatula* (Haliday, 1839).

– Mandible with only three teeth, without a fourth small outgrowth tooth on anterior edge; surface of metapleuron almost smooth and strongly shining; second tergite of metasoma with distinct longitudinal striation only on about its basal half→*L. rupestris* Griffiths, 1968.

5. Antenna with 27 segments (Figure 3O); surface of mesoscutum, propodeum and metapleuron largely glabrous (Figure 3B–D); pterostigma slender, 9.3 times longer than its maximum width, vein m-cu distinctly antefurcal (Figure 3A); first tergite 1.5 times longer than its apical width (Figure 3E)→*L. glabella* sp. nov.

– Antenna with 30–31 segments; surface of mesoscutum, propodeum, and metapleuron with extensive pubescence; pterostigma less slender, 7.0 times longer than its maximum width; vein m-cu more or less interstitial; first tergite 1.2–1.3 times longer than its apical width→*L. luzulae* Godfray, 2023.

*Laotris glabella* sp. nov. (Figure 2 and Figure 3) New Species in China

Zoobank: urn:lsid:zoobank.org:act:9DEAB535-D83B-480D-BD31-7917587997D1

Material examined: Holotype, China. 1♀ (ZJUH), “Qianggangling, Helan Mountain, Aguqi, Inner Mongolia, 3 August 2010, Zengjie, No. 202420006.

Description: ♀; length of body 2.4 mm (excluding ovipositor; Figure 2), of fore wing 2.55 mm.

Head: Transverse (Figure 2, width of head 1.7 times its lateral length in dorsal view (Figure 3I); antenna with 27 segments, respectively (Figure 3O). Third segment (including annellus) 1.3 times longer than fourth segment; length of third, fourth, and penultimate segments 4.0, 3.0 and 2.5 times their width, respectively (Figure 3H); maxillary palp incomplete; eye in dorsal view about 0.9 times as long as temple (Figure 3I); eye in lateral view 2.0 times higher than wide (Figure 3K); frons largely smooth; vertex and temple smooth (Figure 3I); ocelli wide, OOL: diameter of ocellus: POL = 16:10:14; face 1.7 times wider than high, clypeus smooth, distinctly protruding in lateral view, ventro-lateral corners of clypeus rather acute(Figure 3J); mandible parallel-sided, with a minute fourth tooth as an outgrowth present on the ventral side of long and acute second tooth, first and third teeth normal-shaped; median length of mandible 1.5 times as long as its maximum width (Figure 3L–N).

Mesosoma: Length of mesosoma 1.7 times its height in lateral view (Figure 3B); side of pronotum smooth except for some short and regular crenulate posteriorly; dorsal half of epicnemial area almost smooth, and ventral half with deep and narrow crenulae; precoxal sulcus complete and narrowly crenulate, extending to border of metapleuron; pleural sulcus smooth; episternal scrobe round and deep; metapleural flange weakly developed; metapleuron densely rugose posteriorly, but smooth at anterior, only marginal area with sparse setae (Figure 3B); mesoscutum smooth and almost glabrous, only few setae present alongside incomplete notauli; medio-posterior depression deep and elongate at half of mesoscutum; notauli crenulated up to half of mesoscutum (Figure 3C); scutellar suture wide, with three distinct longitudinal carinae and some short rugae laterally; scutellum moderately convex, glossy and smooth (Figure 3C); surface of propodeum densely and irregularly rugose, mid-longitudinal carina and areola absent (Figure 3D).

Wings (Figure 3A): Pterostigma elongate and more or less parallel-sided, 9.3 times longer than its maximum width. vein r issued from anterior quarter of pterostigma, 1.3 times as long as the width of pterostigma; 1-CU1:2-CU1 = 5:14; vein m-cu distinctly antefurcal; first subdiscal cell closed; vein 3-CU1 longer than CU1b.

Legs: Hind coxa smooth (Figure 3F), without ventro-basal tubercle, distinctly larger than first and middle coxa; length of femur, tibia, and basitarsus of hind leg 5.1, 11.7, and 7.1 times their width, respectively; tarsal claws small and slender, slightly shorter than arolium.

Metasoma: First tergite of metasoma 1.5 times its apical width, approximately parallel-sided posteriorly (Figure 3E); surface of first tergite largely glabrous, with irregular rugose and few indistinct longitudinal carinae, lateral carinae not converging; dorsope deep, medium-sized; anterior one-third of second tergite covered with longitudinal striae (Figure 3E); ovipositor does not project beyond apical tergite in retracted position (Figure 3G).

Color: Blackish (Figure 2); labrum, mandibles, legs (except coxae) brown; palps yellowish-brown. coxae, posterior part of the tibia, and whole tarsal segments dark brown; pterostigma and anterior part of veins brown, the remainder of veins and wing membrane hyaline.

Biology: Unknown.

Comparative diagnosis: The new species differs morphologically with *L*. *luzulae* Godfray, 2023 mainly by having 27 antennal segments (vs. 30–31 in *L*. *luzulae*); surface of mesoscutum, propodeum and metapleuron largely glabrous (vs. extensive pubescent in *L. luzulae*); pterostigma slender, vein m-cu distinctly antefurcal (vs. interstitial); first tergite 1.5 (vs. 1.2–1.3) times longer than its apical width.

Etymology: Named “*glabella*” because of the mesoscutum, propodeum and metapleuron are largely glabrous; “*glaber*” is Latin for “bare”.

*Laotris aethidentata* sp. nov. (Figure 4 and Figure 5) New Species in China

Zoobank: urn:lsid:zoobank.org:act:05E7B3D1-826B-4869-9F5F-7545EA01D376

Material examined: Holotype, China. 1♀ (ZJUH), “Liaoning, Shenyang, June–July 1995, Lou Juxian, No. 960299”; Paratype, 1 ♀ (ZJUH), id, but 960301.

Description: ♀, length of body 2.9 mm (excluding ovipositor; Figure 4), of fore wing 2.7 mm.

Head: Transverse (Figure 5I), width of head 1.8 times its lateral length in dorsal view (Figure 5I); antenna with 27 segments, respectively (Figure 5H); third segment (including annellus) 1.4 times longer than fourth segment, length of third, fourth and penultimate segments 3.7, 2.7 and 2.5 times their width, respectively (Figure 5O); length of maxillary palp 0.6 times the height of head; eye in dorsal view about 0.8 times as long as temple (Figure 5I); eye in lateral view 1.6 times higher than wide (Figure 5K); frons, vertex, and temple smooth (Figure 5I); ocelli wide, OOL: diameter of ocellus: POL = 13:8:12; face 1.65 times wider than high, face punctate, covered with extensive and remote long setae alongside eye margin, setae ventrally directed; clypeus smooth, distinctly protruding in lateral view, ventro-lateral corners of clypeus rather acute (Figure 5J); mandible short, without small tooth on second tooth, third tooth small and apex rounded, connected with an inconspicuous fourth tooth, all teeth directed outward; length of mandible equal to its maximum width (Figure 5L,M).

Mesosoma: Length of mesosoma 1.8 times its height in lateral view (Figure 5C); side of pronotum smooth except for some crenulate ventrally; epicnemial area with deep and wide crenulae; precoxal sulcus crenulate, extending almost to middle coxa; pleural sulcus smooth, with some crenulae ventrally; episternal scrobe round and deep; metapleural flange weakly developed; metapleuron sculptured, covered with long setae (Figure 5C); notauli long, narrowly sculptured, almost reaching medio-posterior depression, medio-posterior depression deep and widely crenulated, elongate and up to basal half of mesoscutum; surface of mesoscutum covered with long setae (Figure 5D); scutellar suture narrow, with several distinct longitudinal carinae and short rugae laterally, 4.8 times as long as its maximum width; scutellum moderately convex, glossy and smooth (Figure 5D); surface of propodeum densely and irregularly rugose, mid-longitudinal carina and areola absent, with fine setae and extensively setose laterally (Figure 5E).

Wings (Figure 5A,B): Pterostigma comparatively wide, not parallel-sided, and 4.7 times longer than its maximum width. vein r issued from anterior third of pterostigma, 0.9 times longer than width of pterostigma; vein cu-a slightly postfurcal; vein m-cu distinctly antefurcal; first subdiscal cell closed; vein 3-cu1 longer than cu1b.

Legs: Hind coxa smooth (Figure 5G), without ventro-basal tubercle, slightly larger than first and middle coxa; length of femur, tibia, and basitarsus of hind leg 4.1, 9.0, and 7.3 times their width, respectively; tarsal claws small and slender, slightly shorter than arolium.

Metasoma: First tergite of metasoma 0.9 times its apical width, approximately parallel-sided posteriorly (Figure 5F); surface of first tergite covered with setae, irregularly rugose and strongly sculptured, lateral carinae basally inconspicuous; dorsope deep, medium-sized; surface of second tergite entirely with longitudinal striae; ovipositor projects beyond apical tergite in retracted position, slightly shorter than hind basitarsus (Figure 5N).

Color: Blackish (Figure 4); mandibles and ventral side of metasoma brown; labrum, palps, and legs yellowish brown; pterostigma and anterior part of veins brown, the remainder of veins and wing membrane hyaline.

Biology: Unknown.

Comparative diagnosis: The new species differs morphologically from *L. striatula* Haliday mainly by having a short mandible, its median length as long as width (vs. 1.5 times in *L. striatula*), the second tooth without a small outgrowth tooth (vs. present in *L. striatula*), third tooth small and apex rounded, connected with an inconspicuous fourth tooth, all teeth directed outward (vs. normal and with small third tooth, all teeth directed straightforward in *L. striatula*); pterostigma wide, 4.7 times as long as its width (vs. 5.5 times in *L. striatula*); ovipositor projecting beyond apical tergite in retracted position (vs. not projecting beyond apex in *L. striatula*).

Etymology: Named “*aethidentata*” because of the short mandible and strange third and fourth teeth: “*dentis*” is Latin for “tooth”, and “*aethes*” is Greek for “strange”.

*Victorovita* Tobias, 1985

*Victorovita* Tobias, 1985: 1407; Tobias, 1986: 170; 1998: 300; Perepechayenko, 2000: 72; Godfray & van Achterberg, 2024: 61. Type-species *Victorovita genalis* Tobias, 1985.

**Figure 6:**
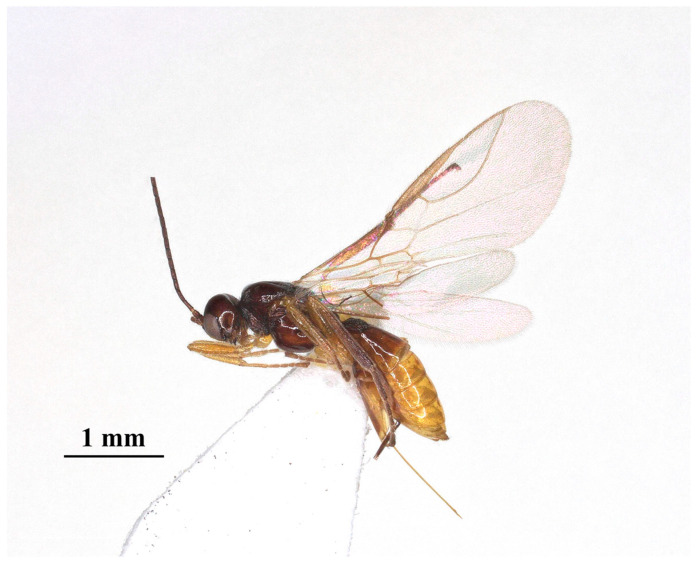
*Victorovita aequalis* sp. nov., ♀, holotype. habitus, lateral aspect.

**Figure 7:**
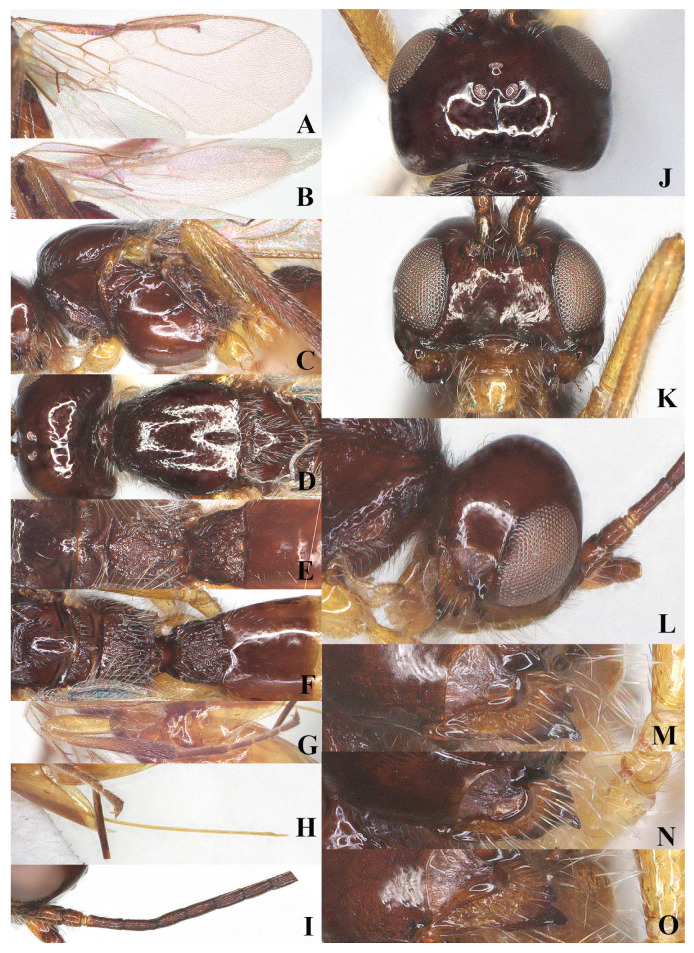
*Victorovita aequalis* sp. nov., ♀, holotype (**A**) fore wing; (**B**) hind wing; (**C**) mesosoma, lateral aspect; (**D**) mesosoma, dorsal aspect; (**E**) propodeum, dorsal aspect; (**F**) metasoma, dorsal aspect; (**G**) hind leg, lateral aspect; (**H**) ovipositor, lateral aspect; (**I**) antenna, lateral aspect; (**J**) head, dorsal aspect; (**K**) head, anterior aspect; (**L**) head, lateral aspect; (**M**) mandible, full view of First and second tooth; (**N**) mandible, full view of second tooth and ventral lamella; (**O**) mandible, full view of ventral lamella.

**Figure 8:**
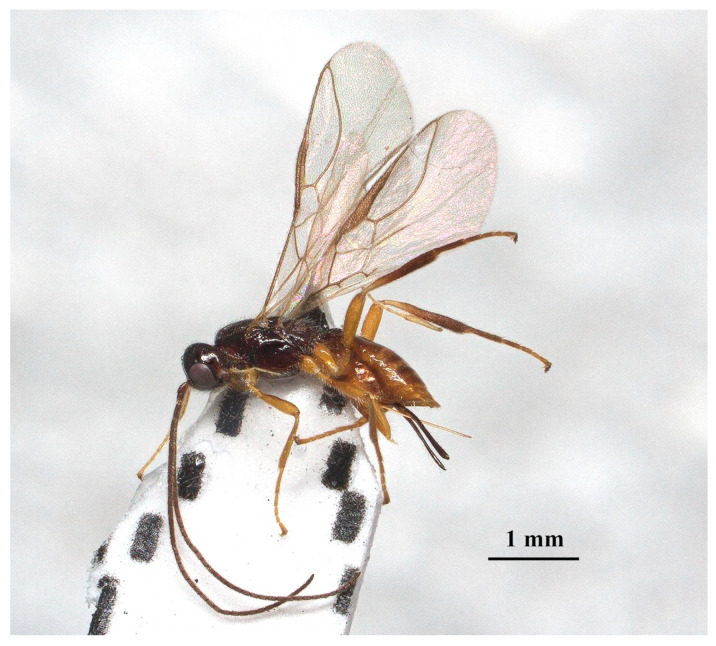
*Victorovita caudata* (Szépligeti, 1901). ♀, China, Ningxia. habitus, lateral aspect.

**Figure 9:**
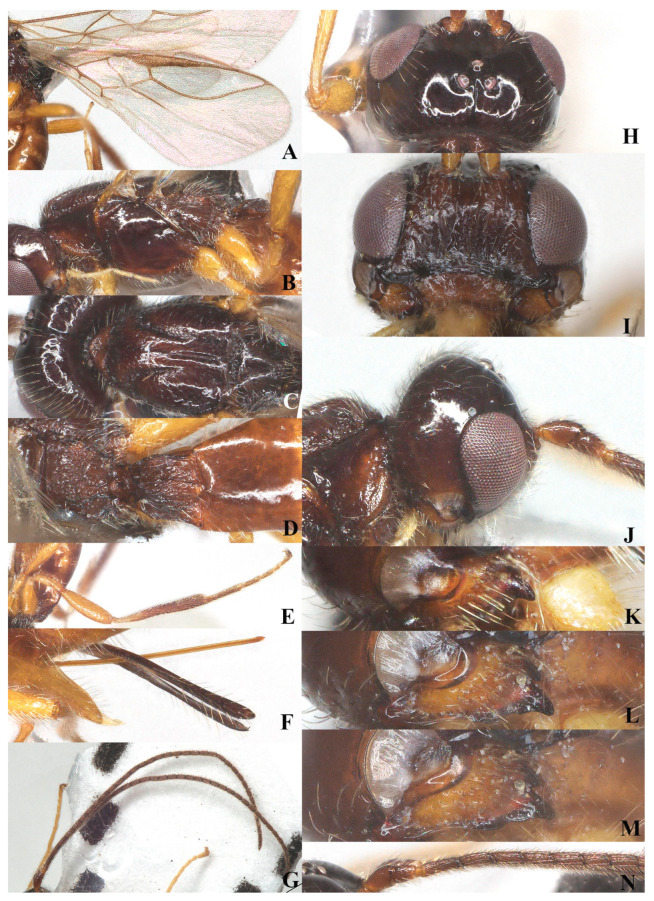
*Victorovita caudata* (Szépligeti, 1901). ♀, China, Ningxia (**A**) wings; (**B**) mesosoma, lateral aspect; (**C**) mesosoma, dorsal aspect; (**D**) propodeum and metasoma, dorsal aspect; (**E**) hind leg, lateral aspect; (**F**) ovipositor, lateral aspect; (**G**) antenna, lateral aspect; (**H**) head, dorsal aspect; (**I**) head, anterior aspect; (**J**) head, lateral aspect; (**K**) mandible, full view of first and second tooth; (**L**) mandible, full view of second tooth; (**M**) mandible, full view of lamella; (**N**) basal segments of antenna, lateral aspect.

Diagnosis: Body dark brown (Figure 6, Figure 7, Figure 8 and Figure 9). Temple subparallel-sided (Figure 7H and Figure 9H); eye glabrous, as long as or slightly shorter than temple in dorsal view (Figure 7H); clypeus very wide and extremely flat, fan-shaped (Figure 7J and Figure 9J); mandibles very short, with only two distinctly developed teeth, first tooth of mandible medium-sized, median tooth long and with narrow ventral lamella (Figure 7K–M); maxillary palpus 5–6 segmented, labial palpus 4 segmented; third segment (including annellus) 1.3–1.4 times longer than fourth segment (Figure 7N and Figure 9N); mesosoma elongate; notauli deep and long, present at basal two-thirds of mesoscutum; medio-posterior depression deep and elongate at posterior two-thirds of mesoscutum, but shallow anteriorly (Figure 7C and Figure 9B); surface of propodeum densely and coarsely reticulate-rugose (Figure 7D and Figure 9D); pterostigma elongate; vein r issuing from anterior of pterostigma; m-cu slightly antefurcal to interstitial; first subdiscal cell closed (Figure 7A and Figure 9A); hind coxa distinctly elongate (Figure 7E and Figure 9E); first tergite of metasoma with irregular rugae and longitudinal carinae, slightly shorter than or as long as its apical width (Figure 7D and Figure 9D); second and following tergites smooth; ovipositor sheath equal to or slightly shorter than hind tibia (Figure 7F and Figure 9F).

Biology: Unknown.

Distribution: Eastern Palearctic, Western Palaearctic.

Key to species of *Victorovita* Tobias, 1985.

1. Pterostigma and veins not pigmented, hyaline; mesoscutum as long as wide; anterior half of propodeum nearly smooth and lustrous, sculptured in posterior half; metasoma narrow, fusiform; hind tarsus distinctly longer than tibia; hind tibia with sparse and smoothened rasp-shaped sculpture, nearly smooth, lustrous; apex of ovipositor sheath with rosette of sparse and very long setae; antenna only with 23 segments; body length 1.7 mm→*V. minuta* Perepechayenko, 2009.

– Pterostigma and veins brown or pale brown (Figure 7A and Figure 9A); mesoscutum 1.2 times as wide as long; surface of propodeum rather coarsely and irregularly rugose (Figure 7E and Figure 9D); metasoma wide, nearly oviform; length of hind tarsus as long as hind tibia (Figure 7G and Figure 9E); hind tibia with dense, rasp-shaped sculpture, weakly lustrous or matte; setae on sheath of ovipositor subequal in length; antenna with 32–39 segments; body larger, body length 2.4–3.4 mm→2.

2. Length of vein 1-R1 of fore wing 0.7 times distance to wing apex (Figure 7A); length of the third segment of antenna 3.1 times its width (Figure 7I); fourth-sixth metasomal tergites brownish yellow (Figure 6); medio-posterior depression of mesoscutum medium-sized (Figure 7D); mandible with a full view of second tooth nearly straight→*V. aequalis* sp. nov.

– Length of vein 1-R1 of fore wing 0.9 times distance to wing apex (Figure 9A); length of the third segment of antenna 3.5 times its width (Figure 9N); fourth-sixth metasomal tergites largely brownish (Figure 8); medio-posterior depression of mesoscutum long (Figure 9C); mandible with full view of second tooth distinct curved ventrally→*V. caudata* (Szépligeti, 1901).

*Victorovita aequalis* sp. nov. (Figure 6 and Figure 7) New species in China

Zoobank: urn:lsid:zoobank.org:act:9146D65E-6C4A-4EA4-B150-9EA4086CF616

Material examined: Holotype, China. 1♀ (ZJUH), “Tibet, Zhixiang Xian, 3–4 July 2013, Liu Zhen, No. 20243714”.

Redescription: ♀, length of body 2.9 mm (excluding ovipositor; Figure 6), of fore wing 3.3 mm.

Head: Transverse (Figure 7H), width of head 1.5 times its lateral length in dorsal view (Figure 7H); antenna incomplete, with nine segments remaining (Figure 7G). segment (including annellus) 1.3 times longer than fourth segment; length of third and fourth segments 3.1 and 2.9 their width, respectively (Figure 7G,N); length of maxillary palp 1.3 times height of head; eye in dorsal view about 0.9 times as long as temple (Figure 7H); eye in lateral view 1.6 times higher than wide (Figure 7J); frons largely smooth; vertex and temple smooth (Figure 7H); OOL: diameter of ocellus: POL = 14:7:8; face 1.4 times wider than high, rather smooth and with long setae; clypeus very wide and extremely flat, fan-shaped, 0.9 times as long as face and 2.5 times its median width (Figure 7I); mandible very short, with only two distinctly developed teeth, first tooth of mandible medium-sized, median tooth long (both teeth pointing downward) and with narrow ventral lamella (Figure 7K–M).

Mesosoma: Length of mesosoma 1.3 times its height in lateral view (Figure 7B); medio-anteriorly pronotum inconspicuously rugose, the remainder of pronotum smooth (Figure 7B); epicnemial area slightly rugose and crenulate ventrally; precoxal sulcus absent; pleural sulcus smooth; episternal scrobe round and small; metapleuron with long setae and roundly protruding medially (Figure 7B); mesoscutum smooth, with dense setae; medio-posterior depression deep and elongate at one-thirds of mesoscutum; notauli long and deep, present two-thirds of mesoscutum, nearly connected with medio-longitudinal depression (Figure 7C); scutellum, mesopleuron, and metapleuron smooth, scutellar sulcus narrow, with one median and two longitudinal carina and some rugae laterally, sulcus 4.0 times wider than its maximum length; surface of propodeum densely and coarsely reticulate-rugose, areola absent, only with two longitudinal carinae at posterior 0.3 of propodeum (Figure 7D).

Wings (Figure 7A): Pterostigma slender, narrow triangular, 6.4 times longer than its maximum width, vein r issued from anterior third of pterostigma, 0.8 times as long as width of pterostigma; 1-SR + M sinuate; 1-CU1:2-CU1 = 5:19; 3-CU1 longer than CU1b; m-cu interstitial; first subdiscal cell 2.3 times as long as its maximum width.

Legs: Hind coxa rather smooth (Figure 7E), without ventro-basal tubercle, rather elongated, longer than fore and middle coxae, length of femur, tibia, and basitarsus of hind leg 3.7, 7.5, and 6.7 times their width, respectively; tarsal claws moderately robust, shorter than arolium.

Metasoma: First tergite widened posteriorly, with irregular rugae and longitudinal carinae; lateral longitudinal carinae not converging; length of first tergite 0.9 times its apical width (Figure 7D); dorsope large and distinct (Figure 7D); total visible length of ovipositor sheath 0.3 times as long as fore wing and 1.1 times as long as hind tibia (Figure 7F).

Color: Dark brown (Figure 6); mandible and clypeus brownish, legs (except apical two-thirds of hind tibia and tarsus brown) and metasoma (except first and second tergites) brownish yellow; palpi yellow; ovipositor sheath brown; pterostigma and anterior part of veins brown, remainder of veins and wing membrane hyaline.

Biology: Unknown.

Comparative diagnosis: The new species is very similar to *V. caudata* (Szépligeti, 1901) but differs morphologically by having the length of vein 1-R1 of fore wing 0.7 times as long as the distance to wing apex (vs. 0.9 times in *V. caudata*); medio-posterior depression of mesoscutum medium-sized (vs. long in *V. caudata*); third segment of antenna 3.1 times its width (vs. 3.5 times in *V. caudata*); fourth-sixth metasomal tergites brownish yellow (vs. dark brown in *V. caudata*).

Etymology: Named “*aequalis*” because the new species looks very similar to *V. caudata* Tobias: “*aequalis*” is Latin for “same”.

*Victorovita caudata* (Szépligeti, 1901) (Figure 8 and Figure 9) New Record in China

*Dacnusa caudata* Szépligeti, 1901: 155.

*Victorovita caudata*; Papp, 2004: 166 (synonymy); Godfray & van Achterberg, 2024: 61.

*Victorovita genalis* Tobias, 1985: 1407; 1986: 170; Perepechayenko, 2009: 81.

Materials examined: China. 2♀ (ZJUH), Ningxia, Liupan Mountain, 3–14 July 2009, Chen Huayan, No. 202210590, 202204982; 2♀ (ZJUH), id. but Zheng Dawei, No. 202207652, No. 202301883.

Redescription: ♀, length of body 2.9–3.1 mm (excluding ovipositor; Figure 8), of fore wing 3.3–3.5 mm.

Head. Transverse (Figure 9H): width of head 1.6–1.7 times its lateral length in dorsal view (Figure 9H); antenna with 37 segments (Figure 9G); antenna slightly longer than body; third segment (including annellus) 1.3–1.4 times longer than fourth segment, length of third, fourth and penultimate segments 3.5, 3.0–3.3 and 2.5–2.6 times their width, respectively (Figure 9G,N); length of maxillary palp 1.2 times the height of head; eye in dorsal view about 0.9 times as long as temple (Figure 9H); eye in lateral view 1.5–1.6 times higher than wide (Figure 9J); frons largely smooth; vertex and temple smooth (Figure 9H); OOL: diameter of ocellus: POL = 12:5:5; face 1.4 times wider than high, rather smooth and with long setae; clypeus very wide and extremely flat, fan shaped, 0.8 times as long as face and 2.4 times its median width (Figure 9I); mandibles very short, with only two distinctly developed teeth, first tooth of mandible medium-sized, median tooth long and with narrow ventral lamella (Figure 9K–M).

Mesosoma: Length of mesosoma 1.5–1.6 times its height in lateral view (Figure 9B); medio-anteriorly pronotum inconspicuously rugose, remainder of pronotum smooth (Figure 9B); epicnemial area slightly rugose and crenulate ventrally; precoxal sulcus absent; pleural sulcus smooth; episternal scrobe round and small; metapleuron with long setae and obtusely protruding medially (Figure 9B); mesoscutum smooth, with dense setae; medio-posterior depression deep and elongate at posterior two-thirds of mesoscutum, but shallow anteriorly; notauli long and deep, present on basal two-thirds of mesoscutum, nearly connected with medio-longitudinal depression (Figure 9C); scutellum, mesopleuron and metapleuron smooth, scutellar sulcus narrow, with one median and two longitudinal carina and some rugae laterally, sulcus 4.0 times wider than its maximum length; surface of propodeum densely and coarsely reticulate-rugose, areola absent, only with two longitudinal carinae at posterior 0.3 of propodeum (Figure 9D).

Wings (Figure 9A): Pterostigma slender, narrow triangled, and 6.4–6.7 times longer than its maximum width. vein r issuing from anterior of pterostigma, 0.7–0.8 times as long as width of pterostigma; 1-SR + M sinuate; 1-CU1:2-CU1 = 5:18–20; 3-CU1 longer than CU1b; m-cu slightly antefurcal to interstitial; first subdiscal cell 2.2–2.4 times as long as its maximum width.

Legs: Hind coxa rather smooth (Figure 9E), without ventro-basal tubercle, rather elongated, and longer than fore and middle coxa. length of femur, tibia, and basitarsus of hind leg 3.6, 10.0, and 6.3 times their width, respectively; tarsal claws moderately robust, shorter than arolium.

Metasoma: First tergite widened posteriorly, with irregular rugose and longitudinal carinae; lateral longitudinal carinae converging medially or subparallel; length of first tergite 0.8–0.9 times its apical width (Figure 9D); dorsope large and distinct (Figure 9D); total visible length of ovipositor sheath 0.3 times as long as fore wing, and 0.8–0.95 times as long as hind tibia (Figure 9F).

Color: Dark brown (Figure 8); mandible and clypeus brownish yellow; legs (except apical two-thirds of hind tibia and tarsus brown) yellowish brown; palpi yellow; ovipositor sheath brown; pterostigma and anterior part of veins brown, the remainder of veins and wing membrane hyaline.

Variation: Body length (excluding ovipositor) 2.9–3.1 mm, of fore wing 3.3–3.5 mm; antennal segments of ♀ 38 (2), 39 (1); total visible length of ovipositor sheath 0.3 times as long as fore wing, and 0.8–0.95 times as long as hind tibia.

Biology: Unknown.

*Coloneura* Foerster, 1863

*Coloneura* Foerster, 1863: 276; Griffiths, 1964: 862; Shenefelt, 1974: 1079; van Achterberg, 1976: 186–192; Tobias, 1986: 212, 1998: 316; Perepechayenko, 2000: 73–74; Godfray & van Achterberg, 2024: 43. Type-species: *Coloneura stylata* Foerster, 1863.

*Isomerista* Foerster, 1863: 275. Type-species: *Isomerista oligomera* Foerster, 1863 [=*Coloneura stylata* Foerster, 1863]. Synonymized by Griffiths, 1964: 862.

*Trisisa* Foerster, 1863: 275. Type-species: *Trisisa exilis* Foerster, 1863 [=*Coloneura stylata* Foerster, 1863]. Synonymized by Griffiths, 1964: 862.

*Merites* Nixon, 1943: 28. Type-species: *Merites taras* Nixon, 1943 [=*Coloneura stylata* Foerster, 1863]. Synonymized by Griffiths, 1964: 862.

*Priapsis* Nixon, 1943: 31; Griffiths, 1964: 862; Shenefelt, 1974: 1079; van Achterberg, 1976: 186–192; Tobias, 1986: 212, 1998: 316. Type-species: *Priapsis dice* Nixon, 1943. Synonymized by Griffiths, 1968a: 11

**Figure 10:**
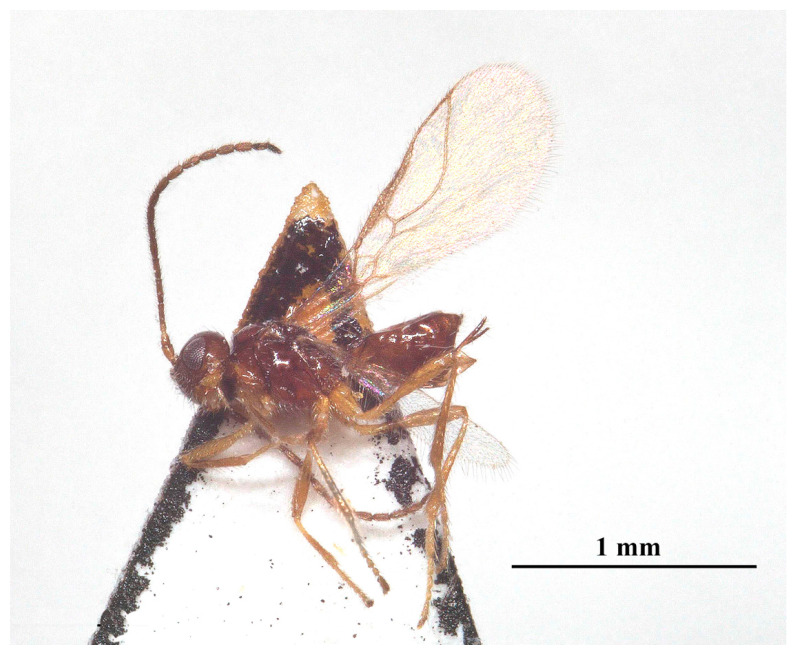
*Coloneura stylata*, Foerster. ♀, China, Zhejiang. habitus, lateral aspect.

**Figure 11:**
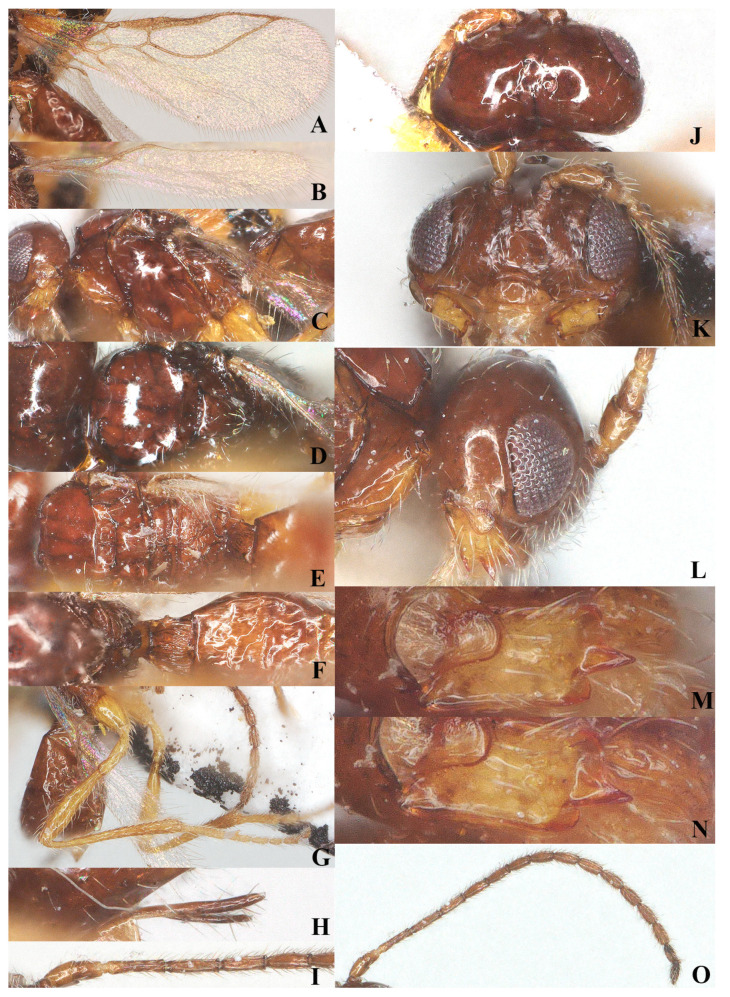
*Coloneura stylata* Foerster. ♀, China, Zhejiang. (**A**) fore wing; (**B**) hind wing; (**C**) mesosoma, lateral aspect; (**D**) mesosoma, dorsal aspect; (**E**) propodeum, dorsal aspect; (**F**) metasoma, dorsal aspect; (**G**) hind leg, lateral aspect; (**H**) ovipositor, lateral aspect; (**I**) basal segments of antenna, lateral aspect; (**J**) head, dorsal aspect; (**K**) head, anterior aspect; (**L**) head, lateral aspect; (**M**) mandible, full view of first and second tooth; (**N**) mandible, full view of third tooth; (**O**) antenna, lateral aspect.

Diagnosis: Body brown (Figure 10). Eyes glabrous; clypeus smooth (Figure 11K); maxillary palpi short, shorter than height of head; mandible with three comparatively slender teeth (Figure 11M,N); first subdiscal cell distinctly open apico-posteriorly and vein CU1b absent (Figure 11A); medio-posterior depression of mesoscutum almost always absent (Figure 11D); metapleural pubescence virtually absent or present but does not form a rosette; metanotum slightly protruding (Figure 11C); dorsope distinctly developed (Figure 11F); second and following tergites smooth (Figure 11F); ovipositor sheath short (Figure 11H).

Biology: Parasitoid of Agromyzidae larvae [7].

Distribution: Eastern Palaearctic, Western Palaearctic.

*Coloneura stylata* Foerster, 1863 (Figure 10 and Figure 11) New Record in China

*Coloneura stylata* Foerster, 1863: 276, Griffiths, 1964: 884, 1968a: 14; van Achterberg, 1976: 189–190; Godfray & van Achterberg, 2024: 43.

Isomerista oligomera Foerster, 1863: 275.

*Trisisa exilis* Foerster, 1863: 275

*Merites taras* Nixon, 1943: 28, 1954: 287.

Material examined: China. 1♀ (ZJUH), Zhejiang, Tianmu Mountain, 22 November 1998, Zhao Mingshui, No. 200001307.

Redescription: ♀, length of body 1.2 mm (excluding ovipositor; Figure 10), of fore wing 1.4 mm.

Head: Transverse (Figure 11J), width of head 2.0 times its lateral length in dorsal view (Figure 11J); antenna with 17 segments (Figure 11O), third segment (including annellus) 1.2 times longer than fourth segment, length of third, fourth and penultimate segments 5.0, 4.3 and 2.8 times their width, respectively (Figure 11I,O); maxillary palp short, about as long as height of eye; eye in dorsal view about 0.9 times as long as temple (Figure 11J); eye in lateral view 1.6 times higher than wide (Figure 11L); frons largely smooth; vertex and temple smooth, temple subparallel behind eyes (Figure 11J); ocelli small, OOL: diameter of ocellus: POL = 15:4:8; face 1.6 times wider than high, clypeus smooth and convex, inverted trapezoid, its apical margin straight medially (Figure 11K); mandible slightly widened apically, mandible 1.1 times as long as its maximum width, middle tooth sharp and slender, ventral and dorsal teeth obtuse, wide and lobe-shaped (Figure 11M,N).

Mesosoma: Length of mesosoma 1.2 times its height in lateral view (Figure 11C); side of pronotum smooth except for some short crenulae medio-anteriorly; dorsal half of epicnemial area almost smooth, and ventral half with deep and narrow crenulae; precoxal sulcus crenulate, present at anterior two-thirds of mesopleuron; pleural sulcus smooth; episternal scrobe round and deep; metapleural flange weakly developed; metapleuron smooth, with sparse long setae (Figure 11C); mesoscutum smooth and glabrous; notauli absent, only present near anterior border of mesoscutum, medio-longitudinal depression absent (Figure 11D); scutellar suture wide, with one distinct longitudinal carina medially and some short rugae or carinae laterally; scutellum moderately convex, glossy and smooth; anterior surface of propodeum densely and irregularly rugose, mid-longitudinal absent and its posterior surface with large coarse rugae and incomplete areola (Figure 11E).

Wings (Figure 11A,B): Pterostigma slender, 7.1 times longer than its maximum width. vein r issued from anterior third of pterostigma, 0.7 times as long as width of pterostigma; 1-CU1:2-CU1 = 1:2; m-cu far antefurcal.

Legs: Hind coxa smooth (Figure 11G), without ventro-basal tubercle. length of femur, tibia, and basitarsus of hind leg 4.3, 10.0, and 6.7 times their width, respectively; tarsal claws small and slender, slightly longer than arolium.

Metasoma: First tergite widened and convex posteriorly, with irregular rugose and longitudinal carinae; length of first tergite 1.2 times its apical width (Figure 11F); laterope deep, medium-sized, total visible length of ovipositor sheath 0.13 times as long as fore wing, and 0.43 times as long as hind tibia (Figure 11G).

Color: Brownish (Figure 10); mandible, clypeus, palpi, labrum, tegulae, and legs yellowish; pterostigma and anterior part of veins brown, the remainder of veins and wing membrane hyaline.

Biology: Parasitoid of *Liriomyza mesnili* (Agromyzidae) [7].

## 4. Discussion

Dacnusini are important endoparasitoids of leaf-mining Diptera pests (Agromyzidae), indicating a relatively high degree of host specificity and well-developed host-finding abilities [5]. Despite having only half the number of genera compared to Alysiini, Dacnusini contains a similar number of species [3,4]. This may suggest that morphological variation at the generic level is less pronounced in Dacnusini than in Alysiini. In this study, we applied an integrative taxonomy analysis by comprehensively utilizing morphology and molecular analysis. The distance-based species delimitation method ABGD and haplotype network analysis were employed to assess species boundaries, both of which yielded consistent results in dividing all treated *Dacnusini* species into seven potential species or MOTUs. The intraspecific genetic divergence within *Laotris* AVD1600 was only 0–0.008, significantly smaller than the interspecific distances between *Laotris* species (0.045–0.095). Based on morphological and molecular evidence, we identified and confirmed three new species: *Laotris glabella* sp. nov., *Laotris aethidentata* sp. nov., and *Victorovita aequalis* sp. nov. Despite extensive efforts, we could not obtain the COI sequences for *Laotris aethidentata* sp. nov. and *Coloneura stylata* Foerster. Specimens of these two genera were collected in 1995 and 1998, respectively, which may have degraded DNA, resulting in low concentration or high fragmentation, making amplification difficult. Recent studies have demonstrated that gene flow, the ratio of population size to divergence time, geographic distance, and taxonomy ranks may reflect the important impact on species delimitation [41,42]. Therefore, future research on these groups would be enhanced by incorporating more comprehensive genomic data and expanding taxonomic sampling to improve species classification.

## Figures and Tables

**Table 1:** The average K2P genetic distance of interspecies and intraspecies K2P genetic distance ranges (bold numbers) for COI sequences.

	*Laotris* sp. (ACY7573)	*Laotris* sp. (ADV6100)	*Laotris g.*	*Laotris l.* (AEO8807)	*Laotris s.* (AEO8806)	*Victorovita a.*	*Victorovita c.*
*Laotris* sp. (ACY7573)	**0.005–0.005**						
*Laotris* sp. (ADV6100)	0.078	**0**–**0.008**					
*Laotris g.*	0.095	0.052	**NA**				
*Laotris l.* (AEO8807)	0.092	0.047	0.045	**0**–**0**			
*Laotris s.* (AEO8806)	0.085	0.065	0.092	0.072	**0.005**–**0.02**		
*Victorovita a.*	0.136	0.144	0.155	0.149	0.138	**NA**	
*Victorovita c.*	0.117	0.126	0.135	0.13	0.12	0.044	**0**–**0.013**

Note: NA indicates that this species only has one sample, and 0–0 means the genetic distance within samples was 0. *Laotris g.* stands for *Laotris glabella* sp. nov.; *l.* for *L. luzulae*; *s.* for *L. striatula*; *Victorovita a.* for *Victorovita aequalis* sp. nov.; and *c.* for *V. caudata.*

## Data Availability

Both the DNA sequences generated in this study and the publicly available data from the BOLD database are accessible under the corresponding accession numbers provided in Table S1.

## Supplementary Materials

The following supporting information can be downloaded at https://www.mdpi.com/article/10.3390/insects15110835/s1. Table S1: List of specimen information used for this study; Table S2: Pairwise genetic distance under K2P model; Dataset S1: ABGD results based on K2P model; Figure S1: Automatic partition results by ABGD based on COI.
