# Supplementary figures and images for: New Records and New Species of Dacnusini (Hymenoptera: Braconidae, Alysiinae) Based on Morphological and Molecular Evidence
Source: Insects. 2024 Oct 24;15(11):835. doi: 10.3390/insects15110835 (PMC11594563; doi:10.3390/insects15110835)

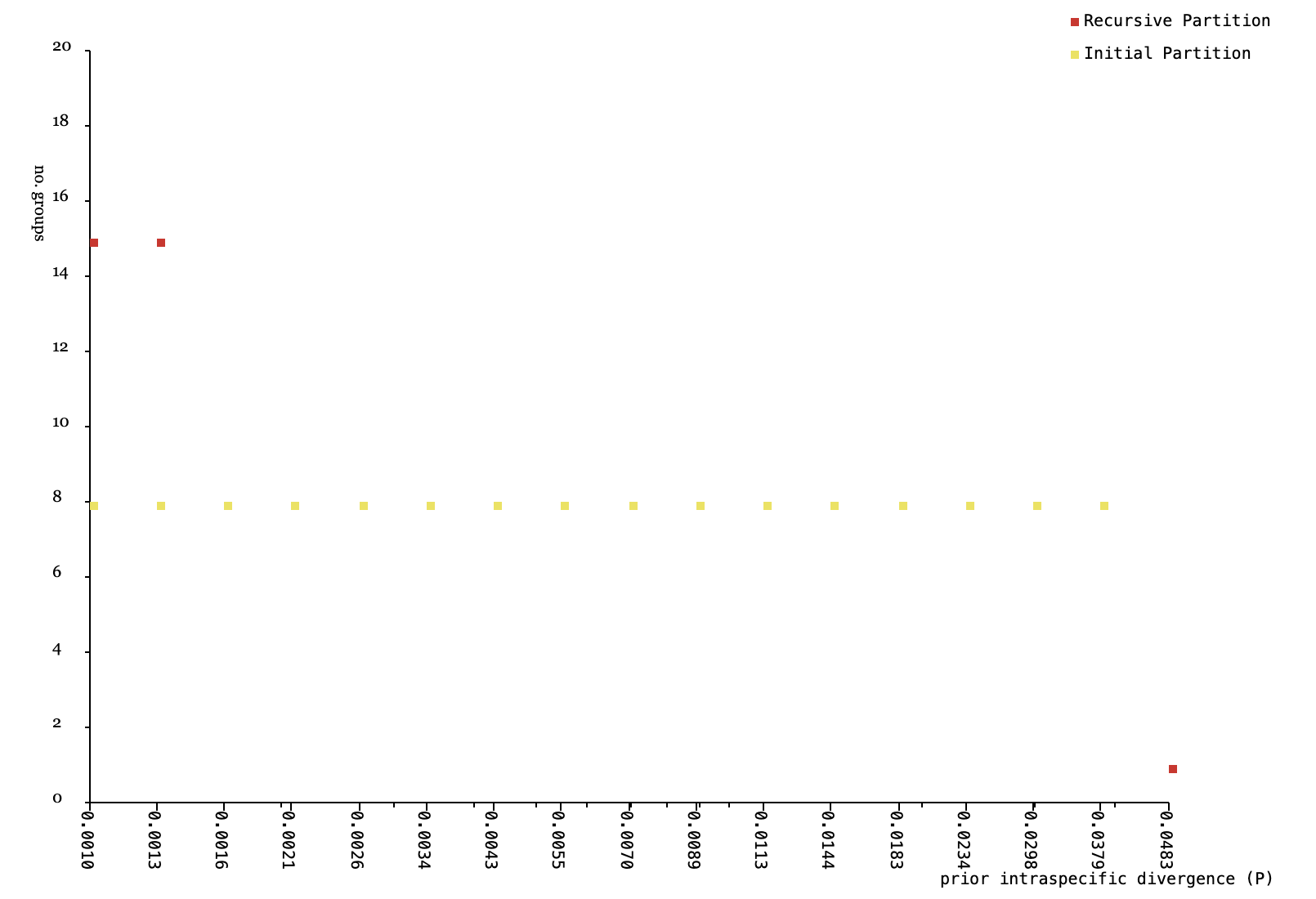

Supplement: Supplementary file 1 [file insects-15-00835-s001.zip › supplementary files/Figure S1 ABGD_K2P_delimitation_partition_results.png]
